# Acute Respiratory Distress Syndrome: Pathophysiology and Therapeutic Options

**DOI:** 10.4021/jocmr761w

**Published:** 2012-01-17

**Authors:** Charalampos Pierrakos, Menelaos Karanikolas, Sabino Scolletta, Vasilios Karamouzos, Dimitrios Velissaris

**Affiliations:** a Intensive Care Department, Brugmann University Hospital, Brussels 1030, Belgium; b Department of Anesthesiology, Washington University School of Medicine, St. Louis, Missouri, USA; c Department of Surgery and Bioengineering, Unit of Cardiothoracic Anesthesia and Intensive Care, University of Siena, Italy; d Department of Internal Medicine, University Hospital of Patras, Rio, Greece; e Department of Anesthesiology and Critical Care Medicine, University Hospital of Patras, Rio, Greece

## Abstract

Acute Respiratory Distress Syndrome (ARDS) is a common entity in critical care. ARDS is associated with many diagnoses, including trauma and sepsis, can lead to multiple organ failure and has high mortality. The present article is a narrative review of the literature on ARDS, including ARDS pathophysiology and therapeutic options currently being evaluated or in use in clinical practice. The literature review covers relevant publications until January 2011. Recent developments in the therapeutic approach to ARDS include refinements of mechanical ventilatory support with emphasis on protective lung ventilation using low tidal volumes, increased PEEP with use of recruitment maneuvers to promote reopening of collapsed lung alveoli, prone position as rescue therapy for severe hypoxemia, and high frequency ventilation. Supportive measures in the management of ARDS include attention to fluid balance, restrictive transfusion strategies, and minimization of sedatives and neuromuscular blocking agents. Inhaled bronchodilators such as inhaled nitric oxide and prostaglandins confer short term improvement without proven effect on survival, but are currently used in many centers. Use of corticosteroids is also important, and appropriate timely use may reduce mortality. Finally, extra corporeal oxygenation methods are very useful as rescue therapy in patients with intractable hypoxemia, even though a survival benefit has not, to this date been demonstrated. Despite intense ongoing research on the pathophysiology and treatment of ARDS, mortality remains high. Many pharmacologic and supportive strategies have shown promising results, but data from large randomized clinical trials are needed to fully evaluate the true effectiveness of these therapies.

**Keywords:**

ARDS; Pathophysiology; Treatment

## Introduction

In 1821, Laennec described a new syndrome characterized by pulmonary edema without heart failure [1]. Subsequently, several terms, such as ”double pneumonia“, ”shock lung“ and ”post traumatic lung“ have been used to describe this syndrome. The term ”Acute Respiratory Distress Syndrome“ was first used in 1967 to describe a distinct clinical entity characterized by acute abnormality of both lungs [2].

In 1994, the American-European Consensus Conference on ARDS established criteria for the diagnosis of ARDS. These criteria include acute onset, bilateral lung infiltrates, no evidence of elevated left atrial pressure and arterial oxygen tension to inspired oxygen fraction (PaO2/FiO2) less than 200. The diagnosis of ARDS requires all these features. However, as these clinical criteria do not always correlate well with diffuse alveolar damage, which is the typical pathologic ARDS feature, ARDS remains a syndrome associated with multiple diagnoses [3], rather than a disease in itself.

Despite substantial progress in understanding ARDS pathophysiology, ARDS remains a major clinical problem, and mortality is still as high as 40 - 46%. The incidence of ARDS in the Intensive Care Unit (ICU) ranges between 4% to 9% depending on patient age and study population [4].

## Pathophysiology

Increased capillary permeability is the hallmark of ARDS. Damage of the capillary endothelium and alveolar epithelium in correlation to impaired fluid remove from the alveolar space result in accumulation of protein-rich fluid inside the alveoli, thereby producing diffuse alveolar damage, with release of pro-inflammatory cytokines, such as Tumor Necrosis Factor (TNF), IL-1 and IL-6 [5]. Neutrophils are recruited to the lungs by cytokines, become activated and release toxic mediators, such as reactive oxygen species and proteases [6]. Extensive free radical production overwhelms endogenous anti-oxidants and causes oxidative cell damage [7].

Inflammation due to neutrophil activation is key in the pathogenesis of ARDS. Fundamental transcription abnormalities, involving NF-kappa B that is required for transcription of genes for many pro-inflammatory mediators, are present in the lungs of ARDS patients [8]. In addition, other factors such as endothelin-1, angiotensin-2 and phospholipase A-2 increase vascular permeability and destroy micro-vascular architecture, enhancing inflammation and lung damage. In conclusion, as several different pathways are involved in ARDS development, no single biomarker can predict outcome in ARDS patients [9].

Computed Tomography studies in the 1980s helped us understand the pathophysiologic alterations in the lungs of ARDS patients [10]. In addition, as lung compliance correlates with the degree of the normally ventilated tissue, lung compliance decreases in ARDS because of decreased lung size, rather than because of lung stiffness, and this hypothesis introduced the concept of “baby lung“ in ARDS [11].

Pulmonary hypertension (PH) is widely recognized as a characteristic feature of ARDS [12]. PH etiology includes parenchymal destruction and airway collapse, hypoxic pulmonary vasoconstriction, presence of other pulmonary vasoconstrictors and vascular compression [13].

The initial phase of fluid accumulation is followed by a proliferation phase characterized by resolution of pulmonary edema, proliferation of type II alveolar cells, fibroblasts and myofibroblasts, and new matrix deposition. This phase starts early (within 72 h) in ARDS, and lasts for more than 7 days. Factors influencing the progression to fibro-proliferation vs. resolution and reconstitution of normal pulmonary parenchymal architecture are poorly understood [14], but patients who develop pulmonary fibrosis exhibit deterioration of pulmonary compliance, progressive hypoxia and ventilator dependence, and increased mortality (> 57%) [15].

Multiple Organ Failure (MOF) is the leading cause of death in ARDS, but the pathophysiologic link between ARDS and MOF is not well defined [16]. However, based on existing data it is not clear whether ARDS is the manifestation of a disease, or it is a disease that causes the MOF syndrome.

## Treatment

Improved understanding of ARDS pathophysiology and advances in technology have introduced new treatments and improved therapeutic strategies. The following paragraphs discuss recent developments in the therapeutic approach to ARDS.

### Low tidal volume ventilation

The concept of ”baby lung“ was introduced in 1980s by Gattinoni et al and generated interest in the use of low tidal volume ventilation as therapeutic strategy in ARDS. Several animal studies showed that ventilation with large tidal volumes and high inspiratory pressures resulted in development of hyaline membranes and inflammatory infiltrates in the lungs, and development of respiratory failure [17].

In the late 1990s four randomized controlled trials (RCTs) evaluated the potential benefit of low tidal volume ventilation in ARDS [18-21]. Although all four studies had limited power, one study by Amato et al [21] demonstrated that the low tidal volume group had higher survival, higher rate of weaning from mechanical ventilation and reduced rate of barotrauma.

Because of conflicting results from these studies, the National Heart Lung and Blood Institute ARDS Network conducted a multicenter RCT on 861 ARDS patients [22], comparing two group of patients ventilated with low vs. high tidal volumes. In-hospital mortality was significantly lower and the number of days without mechanical ventilation was significantly higher in the low tidal volume group. Although this study has been criticized for the high difference of tidal volume between groups, it demonstrated that high tidal volumes should be avoided, and underlined the importance of maintaining low plateau pressures, with 30 cm H_2_O as an acceptable cut-off.

Low tidal volume ventilation is generally well tolerated and it has not been associated with clinically important adverse outcomes, except for hypercapnic respiratory acidosis in some patients. In conclusion, hypercapnia and respiratory acidosis are expected consequences of low tidal volume ventilation. However, there is no evidence that hypercapnia is harmful in ARDS patients, and it may in fact confer some protection against ventilator-associated lung injury.

### PEEP

PEEP is an essential component of mechanical ventilation for patients with ARDS, as it was early observed that PEEP greatly improves oxygenation in ARDS patients. High PEEP levels may open collapsed alveoli and decrease intrapulmonary shunt. Additionally, ventilation-induced alveolar injury is reduced by decreasing alveolar over-distention, because the volume of each subsequent tidal breath is shared by more open alveoli [23]. On the other hand, high PEEP levels may decrease repetitive alveolar opening and closing during the respiratory cycle, thereby promoting lung injury [24].

Three RCTs have evaluated modest vs. high levels of PEEP in patients with ARDS. The NHLB ARDS Network conducted the ALVEOLI trial (Assessment of Low tidal Volume and Elevated end expiratory pressure to Obviate Lung Injury) [25]. This study showed improved PaO2/FiO2 ratio but no benefits with regards to survival or duration of mechanical ventilation in the high PEEP group. Several years later, the Canadian Critical Care Trials Group performed a similar study to determine whether the combination of low tidal volume ventilation with high PEEP could improve mortality to a greater extent compared to low tidal ventilation alone [26]. Results of this study showed reduced need for other rescue therapies such as prone position or NO, but did not show any benefit in survival.

In conclusion, based on published data, high levels of PEEP do not seem to confer any benefit with regards to mortality in ARDS. Because ARDS patients are a heterogeneous population, the apparent absence of benefit from high levels of PEEP could be due to the beneficial effects of high PEEP in some ARDS patients being offset by detrimental effects in other patients [27]. However, data from the RCTs mentioned above suggest that high PEEP levels improve lungs function without any adverse effect on mortality [28].

### Recruitment maneuvers

A recruitment maneuver is a transient increase of trans-pulmonary pressure intended to promote reopening of collapsed alveoli [29]. Techniques described for recruitment maneuvers include sustained high-pressure inflation and increased PEEP, with concurrent reduction of tidal volume [30], but it is not clear if any maneuver is superior to others. Several studies have shown improved gas exchange with recruitment maneuvers, but no RCT has shown benefit on ARDS mortality [31] and a recent systematic review by Fan et al [32] showed that hypotension and decreased saturation occur in 12% and 8% of patients respectively during or after such maneuvers. Based on currently available data, although routine recruitment maneuvers are not recommended in ARDS, such maneuvers can dramatically improve oxygenation in certain patients, and should be considered as rescue therapy in patients with life-threatening refractory hypoxia [33].

### Prone position

Prone positioning has been used in ARDS for over 30 years. In 1976 Piehl et al. reported improved oxygenation in ARDS patients when they were turned to the prone position [34]. Since then, several observational studies on ARDS have found similar results, and improvement in oxygenation can sometimes be dramatic [35]. Mechanisms proposed to explain the observed beneficial effects of prone positioning include increased chest wall elastance decreased compression of lung tissue in the dependent regions and recruitment of alveoli, more homogeneous ventilation due to decreased ventilation-perfusion inequalities and reduced ventilator induced lung injury [36].

Four RCTs have investigated the effect of prone positioning on outcome. The first trial by the Prone-Supine Study group randomized 304 patients with a wide range of severity of acute lung injury [37].Patients remained prone for 7 h/day on average, for up to 10 days, but there was no effect on survival. Three years later, Guerin et al conducted a similar multicenter study [38]: patients remained prone for about 8 h/day, and prone positioning continued until clinical criteria for improvement were met, but this study also did not show a reduction in mortality. Two subsequent RCTs attempted to correct some shortcomings of the earlier study: they only included patients with ARDS, and patients remained prone for most of the day (about 20 h). The first RCT by Mancebo et al was terminated prematurely, after only including 142 patients, because of problems with patient recruitment [39]. A more recent multicenter RCT by Taccone et al, included 344 patients [40], and showed significantly increased frequency of adverse events (airway obstruction, hypotension, vomiting, accidental extubation) in patients treated with prone position. Neither of the last two studies showed any survival benefit using the prone position in patients with severe ARDS.

In conclusion, existing data do not support routine use of the prone position in ARDS. However, because all published studies have shown improved oxygenation, prone positioning is an attractive rescue treatment for ARDS patients with severe hypoxemia, even though a survival benefit has never been demonstrated.

### High-frequency ventilation

The idea of high frequency ventilation (HFV) is to provide tidal volumes below that of anatomic dead space at high frequency (> 60 breaths per minute). Compared to conventional mechanical ventilation, mean airway pressure is higher [41]. Two studies, by Hamilton and Chan, showed reduced risk for barotrauma and lung over-distention, after performing high frequency ventilation [42,43]. High frequency ventilation can be applied by different modes, such as high-frequency percussive ventilation, high-frequency jet ventilation and high-frequency oscillatory ventilation (HFOV) [44]. In the absence of studies showing superiority of one method over another, HFOV is the HFV method used more often in adult critical care [43].

In HFOV very small tidal volumes (1 - 4 ml/kg) are delivered at high frequency (3 - 15 Hz) by an oscillatory pump [45]. However, the use of HFOV as rescue therapy in patients with refractory hypoxia remains controversial. There are two RCTs comparing HFOV with conventional mechanical ventilation. The first RCT by Derdak et al found a trend for decreasing 30-day mortality [46] even though relatively high tidal volume was used in the control group. The second RCT by Bollen et al was terminated prematurely because of slow enrollment, but found an opposite trend in mortality [47]. Two meta-analyses also had conflicting results. The first one included only 2 RCTs comparing HFOV vs. conventional ventilation, and did not find any mortality reduction or improvement of oxygenation [48]. However, a more recent study by Sud et al included 8 RCTs and found reduced 30-day mortality with HFOV compared to conventional ventilation [49]. In conclusion, the role of HFOV in ARDS is not well defined, and deserves further study from well designed RCTs.

### Supportive treatment of ARDS

#### Fluid management

Early data indicate that increased fluid administration is correlated with worse outcome in ARDS, whereas negative fluid balance was associated with better outcome [50]. The ARDS network conducted a RCT with 1000 patients randomized to receive conservative or liberal fluid administration. The conservative strategy group showed improved lung function and shortened duration of mechanical ventilation, but there was no difference in non pulmonary organ failures and 60-day mortality.

#### Transfusions

Allogenic blood transfusion is associated with detrimental immuno-modulatory effects that may result in ARDS [51]. Consequently, conservative transfusion strategies may decrease the incidence of ARDS.

#### Sedation

Several studies have shown that newer ventilation strategies using low tidal volume and high levels of PEEP do not require high doses of sedation [52]. Furthermore, evidence suggests that use of sedatives may increase duration of mechanical ventilation and ICU length of stay and may even be associated with higher mortality [53]. In addition, there is evidence that spontaneously breathing ARDS patients have improved cardiopulmonary function, presumably by recruiting non ventilated lung units [54]. Therefore, based on current evidence, avoidance or minimization of sedation and paralysis is preferred in ARDS.

#### Neuromuscular blocking agents

Administration of neuromuscular blocking agents (NMBA) in addition to sedation can result in improvement in severe hypoxemia, because paralysis improves patient-ventilator synchronism and reduces oxygen consumption [55]. Muscle relaxation may also improve chest wall compliance. NMBAs may also have anti-inflammatory effects that could decrease the inflammation associated with ARDS. However, because there is evidence that NMBAs increase the risk of acquired neuromuscular weakness, thereby making weaning from mechanical ventilation more difficult, and may even increase mortality [56].

#### Nutrition

Two RCTs [57,58] and a meta-analysis by Puntes-Arruda et al [59] showed that administration of enteral nutrition containing high concentrations of eicosapentanoic acid and γ-linoleic acid and ω-3 fatty acids increased oxygenation and decreased ICU stay and 28-day mortality in ARDS.

### Pharmacologic agents

#### Vasodilators

Because diffuse pulmonary vasoconstriction is part of ARDS pathophysiology, selective vasodilatation of well ventilated areas seems an attractive method to improve gas exchange in ARDS patients.

Inhaled nitric oxide (iNO) causes vasodilation of ventilated lung units and redistribution of pulmonary blood flow away from non-ventilated lung areas, without adverse systemic hemodynamic effects. Four RCTs evaluated the effects of iNO and showed transient improvement in oxygenation [60-63], but no improvement in mortality. Similarly, one meta-analysis found transient oxygenation improvement but no survival benefit with iNO [64]. Consequently, iNO may be useful as short-term rescue therapy in patients with severe hypoxemic respiratory failure.

Inhaled prostacycline is another selective pulmonary vasodilator. Importantly, liposomal PGE1 influences neutrophil function and decreases neutrophil accumulation and lung leak. Although inhaled prostacycline improves oxygenation, it has not been shown to reduce duration of mechanical ventilation or mortality in ARDS patients [65]. Despite the lack of sufficient data supporting the use of prostacycline as alternative to iNO, prostacycline is increasingly used as pulmonary vasodilator, because of the high cost of iNO [66].

#### Vasoconstrictors

Vasoconstrictors can improve oxygenation in ARDS patients by decreasing intrapulmonary shunt. Phenylephrine [67] and almitrine [68] have been used in small studies, mainly as adjuncts during administration of NO. B-blockers have also been shown to increase arterial oxygenation in patients with ARDS [69]. However, the role of these agents in ARDS has not been adequately evaluated, and deserves further study.

#### Anti inflammatory agents

The interaction between nuclear factor-kappa B (NF-kB) and glucocorticoid receptor alpha (GRa) is a key mechanism regulating the progression of systemic and pulmonary inflammation in ARDS [70]. The ability of GC-GRa to down-regulate NF-kB activation is critical for the resolution of systemic and pulmonary inflammation in ARDS [71]. Although several studies showed that corticosteroids confer no benefit and may even cause harm [72], corticosteroids are still used in clinical practice.

Timing of corticosteroid administration and duration of therapy may be important, and should be taken into consideration. A RCT conducted by the ARDS Network, randomized 180 patients with persistent (> 7 days) ARDS, to receive methylprednisolone (2 mg/kg/d) or placebo for 21 days, and showed improved oxygenation and more ventilator-free days in the methylprednisolone group, but no significant improvement in mortality [73]. Another RCT evaluated early corticosteroid administration, and showed that methylprednisolone administration (1mg/kg/d) [74] less than 72 after the onset of ARDS reduced mortality. However, these results should be interpreted with caution, because this study included a large number of patients with septic shock.

Conflicting data exist concerning the correlation between corticosteroids and ICU neuromyopathy. A sub-analysis of study survivors did not show any association between randomization to corticosteroids and increased risk of neuromyopathy [75]. In conclusion, the relationship between corticosteroids and ICU neuromyopathy is an important issue that deserves further study [76].

Several other anti-inflammatory factors like Ibuprofen [77], ketoconazole [78], neutrophil elastase inhibitors (ONO 5046) [79], NF-KB inhibitors [80], recombinant soluble complement receptor-1 [81], and liposomal prostaglandin E1 [82] have been evaluated in ARDS patients without success.

#### Beta agonists

There are substantial evidences that b2-agonists may play a potential role in the treatment of patients with ARDS. B2 agonists have been found to have anti-inflammatory effects by direct influence on neutrophil function and by reducing the secretion of several pro-inflammatory cytokines. Additionally, b2-agonists can reduce the endothelial permeability and stimulate the fluid clearance from the lungs [83]. In a small RCT using the thermodilution method (PiCCO), intravenous salbutamol (15 μg/kg/h) use for seven days reduced extravascular lung water compared to placebo [84]. The effects of inhaled b-agonists have not, to this date, been adequately evaluated, but will be investigated in an ongoing study conducted by ARDS network (NCT00434993)

Several other pharmacological agents, including glutathione lisofylline [85], N-Acetylcysteine[86], and surfactant [87] have been evaluated, but none of them has been shown to be effective for treatment of ARDS.

### Extracorporeal techniques

Extracorporeal membrane oxygenation (ECMO) has been studied since 1970s as a method for supporting gas exchange in patients failing conventional ventilation [88]. A RCT conducted in 1979 showed that ECMO use had no effect on long-term survival of ARDS patients [89]. Nowadays, ECMO is used to support oxygenation of patients with severe ARDS, thereby allowing use of decreased ventilator settings (tidal volume, respiratory rate, FiO2), in an attempt to minimize ventilator induced lung injury.

In a more recent study 180 patients with severe ARDS were randomized to support by ECMO vs. conventional treatments. This study showed significantly improved survival (63% vs. 47%, P = 0.03) at 6 months in the ECMO group [90]. Although no definite conclusions can be drawn from this study [91], the results of this study suggest that ECMO can be used as a rescue therapy in cases of very severe ARDS.

Extracorporeal CO2 removal (ECCO2R) is an alternative device that uses veno-venous circuit for removal of CO2 at much lower extracorporeal flow rates compared to ECMO. A RCT conducted before the year 2000 used ECCO2R and showed no effect on mortality [92]. In another study ECCO2R was combined with low frequency positive pressure ventilation (2 - 3 b/min), and showed improvement in lung mechanics [93]. Overall, extracorporeal support technologies produce significant temporary improvement in ARDS patients with severe respiratory dysfunction, but this improvement does not seem to affect outcome. New, well conducted clinical studies are needed to better evaluate the role of ECMO and ECCO2R on survival in ARDS.

## Mortality

Conflicting data exist about the evolution ARDS mortality over time. A meta-analysis by Phua et al did not find any mortality reduction in recent years [94], whereas another meta-analysis by Zambon et al. showed reduced mortality in recent years [4]. In the past, several studies evaluated patterns of ARDS mortality over time within the same institution [95-99], and all studies, except for two [98,99], found decreasing mortality in ARDS. The observed discrepancy between different studies may be due to different investigational methods, but we can conclude that ARDS mortality remains high (41 - 46%). Regardless of improvements in recent years, ARDS mortality is higher in older patients and in medical patients [100]. However, the impact, if any, of newer treatment strategies on ARDS mortality has not been evaluated, because most studies are referred to the period before the year 2000.

## Abbreviations

ARDS: Acute Respiratory Distress Syndrome
ECCO2R: Extracorporeal CO2 Removal
ECMO: Extracorporeal Membrane Oxygenation
HFV: High Frequency Ventilation
HFOV: High Frequency Oscillatory Ventilation
ICU: Intensive Care Unit
IL: Interleukin
iNO: Inhaled Nitric Oxide
MOF: Multiple Organ Failure
NMBAs: Neuromuscular Blocking Agents
PEEP: Positive End Expiratory Pressure
PH: Pulmonary Hypertension
pRBCs: Packed Red Blood Cells
RCT: Randomized Controlled Trial
TNF: Tumor Necrosis Factor
TV: Tidal Volume
